# Lymphoplasmacytic Sclerosing Pancreatitis and Retroperitoneal Fibrosis

**DOI:** 10.1155/2008/719513

**Published:** 2008-03-13

**Authors:** Nigel K. F. Koo Ng, Jin J. Bong, Robin C. Williamson

**Affiliations:** ^1^Department of Surgery, Charing Cross Hospital, London W6 8RF, UK; ^2^Division of Hepatobiliary Surgery, Department of Surgery, Royal Surrey County Hospital, Egerton Road, Guildford, Surrey GU2 7XX, UK; ^3^Department of Surgery, Hammersmith Hospital, London W12 0HS, UK

## Abstract

Although cases of lymphoplasmacytic sclerosing pancreatitis (LSP) associated with idiopathic retroperitoneal fibrosis have been reported, the association is rare. We describe a 74-year-old man who presented with obstructive jaundice and weight loss. Nineteen months earlier, he had been diagnosed with idiopathic retroperitoneal fibrosis and treated with bilateral ureteric stents. Initial investigations were suggestive of a diagnosis of LSP, however, a malignant cause could not be ruled out. He underwent an exploratory laparotomy and frozen sections confirmed the diagnosis of LSP. An internal biliary bypass was performed using a Roux loop of jejunum, and the patient made an uneventful recovery. This case illustrates the difficulty in distinguishing LSP from pancreatic carcinoma preoperatively.

## 1. INTRODUCTION

Lymphoplasmacytic sclerosing pancreatitis (LSP) or autoimmune pancreatitis is a rare condition characterised by diffuse fibroinflammatory infiltrates that can involve both the pancreatic ducts and acinar parenchyma. It is also known as primary inflammatory sclerosis of the pancreas, sclerosing pancreatitis, autoimmune sclerosing pancreatitis, duct destructive chronic pancreatitis, and sclerosing pancreatico-cholangitis [1, 2].

Since the condition was first described in 1961 by Sarles et al., several cases have been reported, and in recent years, LSP has become increasingly recognised as an important and unique cause of chronic pancreatitis. Of particular note, it presents with a pancreatic mass that can be extremely difficult to differentiate from pancreatic carcinoma (which it may resemble both clinically and radiologically). It has been reported that “benign but clinically suspicious” Whipple resections are relatively common in high-volume centers (9.2%) and that LSP represents 23.4% of Whipple resections performed for benign disease [3]. The occasional association of LSP with other autoimmune conditions is well documented [4], including Sjogren's syndrome, primary sclerosing cholangitis, inflammatory bowel disease, and systemic lupus erythematosus. Although cases of LSP associated with idiopathic retroperitoneal fibrosis have also been reported, the association is rare. To our knowledge, not more than 14 cases have been reported in the literature [4–11].

## 2. CASE REPORT

A 74-year-old Afro-Caribbean man presented with a 4-week history of obstructive jaundice, weight loss, and fatigue. He had a background of noninsulin-dependent diabetes and hypertension. Nineteen months earlier, symptoms of polyuria and nocturia had led to a diagnosis of retroperitoneal fibrosis: renal function was impaired, and both kidneys were poorly perfused on CT scan, with evidence of retroperitoneal fibrosis obstructing both ureters. Subsequently, bilateral ureteric stents were inserted.

Since then, the patient had been well until recently developing jaundice. USS showed extrahepatic and intrahepatic biliary dilatation but no gallstones. The pancreas was bulky throughout, although no focal lesion was identified. ERCP showed an irregular stricture in the lower CBD suggestive of malignancy (although the biliary brushings later showed no malignant cells), and an endobiliary stent was inserted. Segments of the pancreatic duct were also dilated. CT confirmed that the pancreas was markedly bulky with no focal mass lesion or abnormal calcifications. There was thickening of soft tissue around the porta hepatis suggestive of lymphadenopathy. Although the main concern was pancreatic carcinoma, LSP was also considered to be a possible diagnosis. An autoimmune screen was negative for ANA, cytoplasmic antibodies (Hep-2), ANCA IIF, mitochondrial antibodies, smooth muscle antibodies, liver/kidney microsomal antibodies, Anti-GPC, rheumatoid factor, and striated muscle antibodies. The IgG level was raised at 27.0 g/L (5.3–16.5 g/L), particularly IgG4 at 15.10 g/L (0.0–1.3 g/L). Although, there have been reports of an association with a raised IgG4 level and LSP [12], the diagnosis in this case was still unclear. To confirm the diagnosis, an exploratory laparotomy was considered to be most appropriate (and preferable to EUS FNA) as it offered the opportunity to obtain multiple biopsies from a diffusely enlarged pancreas, whilst also offering a therapeutic option.

At laparotomy, the entire pancreas was hard, swollen, and actively inflamed, most particularly the body of the gland. The appearances were not typical for pancreatic cancer or chronic pancreatitis. A shave biopsy of a nodule on the body of pancreas plus three core biopsies with a Trucut needle and a fourth from the head of pancreas were submitted to frozen-section histopathology examination; all showed florid chronic inflammation but no cancer. A portion of thickened retroperitoneum tissue in front of the aorta was also removed and this was shown to be benign fibrotic tissue. Since the pathology was consistent with a diagnosis of LSP, an internal biliary bypass was performed using a Roux loop of jejunum. The patient made a good postoperative recovery and was referred to gastroenterology for further management. Steroids were given postoperatively and he was discharged nine days later.

## 3. DISCUSSION

LSP primarily affects middle-aged men, with a mean age of 56 years [13]. There is a male predominance of approximately 2 : 1 [13]. The recently increasing number of articles on LSP is an indication of the current interest in this subject. Its similarity to pancreatic carcinoma, both clinically and radiologically, presents a diagnostic challenge. In distinguishing LSP from pancreatic carcinoma, there are certain suggestive features (see Table 1) [14–16]. The Japanese Pancreas Society proposed diagnostic criteria for LSP in 2002 (see Table 2) [14, 17].

Patients with LSP also have elevated serum IgG4 levels which return to normal after administration of oral steroids. Only a limited number of conditions are associated with elevated IgG4 (such as atopic dermatitis, some parasitic diseases, pemphigus vulgaris, and pemphigus foliaceus). Hamano and associates found high serum IgG4 concentrations specifically in patients with LSP and not in those with “ordinary” chronic pancreatitis, primary biliary cirrhosis, primary sclerosing cholangitis, or Sjogren's syndrome [12]. Not only does this finding suggest that LSP is immunologically different, but it may also follow that IgG4 would be a useful marker [12].

The association of LSP with other autoimmune conditions, including retroperitoneal fibrosis, is well recognised. Our patient developed retroperitoneal fibrosis prior to LSP, but review of the 14 previous cases indicates that the onset of LSP is variable. Further, hydronephrosis secondary to ureteric obstruction (as in our case) was not uncommon [4–11]. The aetiology of retroperitoneal fibrosis is unclear, but the presence of autoantibodies and the response to steroids in some patients may suggest an autoimmune mechanism. Infiltration with IgG4-positive plasma cells has been shown in both the pancreas and the retroperitoneal mass of patients with LSP and retroperitoneal fibrosis [5], indicating a likely common aetiology.

LSP can be treated with medical therapy (steroids in reducing dosage) or surgically. The prognosis is usually good with either form of management but an important factor is the difficulty in diagnosing LSP, which can so often mimic a malignant intra-abdominal mass. Preoperative or even intraoperative diagnosis can be problematic, particularly in patients for whom there are no clinical grounds to suspect LSP. It has been suggested that surgical resection may be an effective treatment for LSP especially when immunological tests are normal or when malignancy is highly suspected (this may be the only option to exclude malignancy) [18]. However, surrounding inflammation can make surgical resection hazardous, so a trial of steroids is worthwhile if a confident diagnosis can be made without laparotomy [18].

## Figures and Tables

**Table 1:** Diagnostic features suggestive of lymphoplasmacytic sclerosing pancreatitis.

	Features suggestive of LSP
(1)	Minimal or only mild symptoms, usually without episodes of acute pancreatitis
(2)	Diffusely irregular narrowing of the main pancreatic duct and occasional stenosis of the intrapancreatic bile duct on ERCP images
(3)	Diffuse enlargement of the pancreas
(4)	Increased levels of serum gamma globulin or IgG
(5)	Presence of autoantibodies
(6)	Fibrotic changes with lymphocyte infiltration in the pancreas
(7)	Occasional association with other autoimmune conditions
(8)	Rare pancreatic calcification or cysts
(9)	Clinical response to steroids

**Table 2:** To make a diagnosis of LSP, criteria *a* must be present, together with criteria *b* and/or *c*.

	Diagnostic criteria for LSP
(a)	Pancreatic imaging studies showing diffuse narrowing of the main pancreatic duct with an irregular wall (more than 1/3 of the length of the entire pancreas) with enlargement of the pancreas
(b)	Abnormally elevated levels of serum gamma globulin and/or IgG, or the presence of autoantibodies
(c)	Histopathological examination of the pancreas shows fibrotic changes with lymphocyte and plasma cell infiltration
